# Anti-inflammatory effects of cold atmospheric plasma irradiation on the THP-1 human acute monocytic leukemia cell line

**DOI:** 10.1371/journal.pone.0292267

**Published:** 2023-10-18

**Authors:** Ito Hirasawa, Haruka Odagiri, Giri Park, Rutvi Sanghavi, Takaya Oshita, Akiko Togi, Katsunori Yoshikawa, Koji Mizutani, Yasuo Takeuchi, Hiroaki Kobayashi, Sayaka Katagiri, Takanori Iwata, Akira Aoki

**Affiliations:** 1 Department of Periodontology, Graduate School of Medical and Dental Sciences, Tokyo Medical and Dental University, Tokyo, Japan; 2 Sekisui Chemical Co., Ltd., Ibaraki, Japan; 3 Department of Lifetime Oral Health Care Science, Graduate School of Medical and Dental Sciences, Tokyo Medical and Dental University, Tokyo, Japan; Kansai Medical University: Kansai Ika Daigaku, Institute of Biomedical Science, JAPAN

## Abstract

Cold atmospheric plasma (CAP) has been studied and clinically applied to treat chronic wounds, cancer, periodontitis, and other diseases. CAP exerts cytotoxic, bactericidal, cell-proliferative, and anti-inflammatory effects on living tissues by generating reactive species. Therefore, CAP holds promise as a treatment for diseases involving chronic inflammation and bacterial infections. However, the cellular mechanisms underlying these anti-inflammatory effects of CAP are still unclear. Thus, this study aimed to elucidate the anti-inflammatory mechanisms of CAP *in vitro*. The human acute monocytic leukemia cell line, THP-1, was stimulated with lipopolysaccharide and irradiated with CAP, and the cytotoxic effects of CAP were evaluated. Time-course differentiation of gene expression was analyzed, and key transcription factors were identified via transcriptome analysis. Additionally, the nuclear localization of the CAP-induced transcription factor was examined using western blotting. The results indicated that CAP showed no cytotoxic effects after less than 70 s of irradiation and significantly inhibited interleukin 6 (*IL6*) expression after more than 40 s of irradiation. Transcriptome analysis revealed many differentially expressed genes (DEGs) following CAP irradiation at all time points. Cluster analysis classified the DEGs into four distinct groups, each with time-dependent characteristics. Gene ontology and gene set enrichment analyses revealed CAP-induced suppression of *IL6* production, other inflammatory responses, and the expression of genes related to major histocompatibility complex (MHC) class II. Transcription factor analysis suggested that nuclear factor erythroid 2-related factor 2 (NRF2), which suppresses intracellular oxidative stress, is the most activated transcription factor. Contrarily, regulatory factor X5, which regulates MHC class II expression, is the most suppressed transcription factor. Western blotting revealed the nuclear localization of NRF2 following CAP irradiation. These data suggest that CAP suppresses the inflammatory response, possibly by promoting NRF2 nuclear translocation.

## Introduction

Physical plasmas, recognized as the fourth state of matter, encompass excited ionized gases that comprise reactive species, atoms, electrons, and other constituents [1]. Recent technological advancements have facilitated the generation of physical plasma at low temperatures and under atmospheric pressure. This variant is commonly referred to as cold atmospheric plasma (CAP). By subjecting biological systems to CAP irradiation, a range of effects, such as cell proliferation, programmed cell death, anti-inflammatory responses, and sterilization, can be induced, primarily through the action of reactive oxygen and nitrogen species (RONS). The potential applications of CAP in diverse medical domains, including wound healing and cancer treatment, have been the subject of extensive investigation [1–3].

Periodontal disease is chronic periodontal tissue inflammation caused by microbiological dysbiosis involving gingival bleeding and loss of alveolar bone [4], and using CAP to treat periodontal diseases is also under consideration. CAP irradiation reportedly suppresses inflammatory cytokines and loss of alveolar bone in rat periodontitis [5], reduces periodontal pathogens, and improves bleeding on probing and the gingival index in a clinical study [6]. The bactericidal and anti-inflammatory effects are presumed to be the mechanisms underlying the improvement of periodontal disease induced by CAP.

RONS produced during CAP treatment destabilize bacterial structures, such as proteins, cellular envelopes, and DNA. This mechanism is widely acknowledged as the primary means by which CAP achieves sterilization [7,8]. However, the cellular mechanism of the anti-inflammatory effects of CAP remains unclear because of its varying effects from pro-inflammatory to anti-inflammatory, depending on the plasma dose and therapeutic target. CAP can suppress inflammation in monocytes [9], *ex vivo* human oral tissue [10], and periodontitis in a rat model [5]. In contrast, it promotes inflammation in keratinocytes [11], dendritic cells [12], and wound healing in a mouse model [13].

The nuclear factor erythroid 2-related factor 2 (NRF2) plays a crucial role in cellular reactions to plasma irradiation. NRF2 is a transcription factor (TF) that exerts cellular protective effects against oxidative stress via expressing antioxidant response element (ARE)-regulated genes. Microarray and proteomic analyses of CAP-treated keratinocytes have suggested NRF2 pathway activation [14]. Furthermore, histological and gene expression analyses confirmed the nuclear migration of NRF2 and ARE-regulated gene activation in a mouse wound model [13]. While the involvement of NRF2 in the plasma effect has been established in certain cell types, its contribution to the anti-inflammatory properties of CAP against immune cells during inflammatory conditions remains unclear. Furthermore, other TFs supporting the widespread activity of CAP have not yet been identified.

Therefore, we aimed to reveal the mechanism underlying the anti-inflammatory effects of CAP on the human acute monocytic leukemia cell line, THP-1, using transcriptome analysis.

## Materials and methods

### Cell culture and lipopolysaccharide (LPS) stimulation

The THP-1 cells (JCRB0112.1 / LOT:11012017, JCRB cell bank, Osaka, Japan) were cultured at 2 × 10^4^ cells per well in a 96-well plate containing 100 μL RPMI 1640 (Fujifilm Wako Pure Chemical Corporation, Osaka, Japan) supplemented with 10% fetal bovine serum (FBS, Thermo Fisher Scientific, Waltham, MA, USA) and 1% penicillin-streptomycin (Fujifilm Wako Pure Chemical Corporation). The cells were incubated at 37°C in a humidified atmosphere containing 5% CO_2_. Cells were stimulated with LPS from *Porphyromonas gingivalis* (Pg, InvivoGen, San Diego, CA, USA) at a final concentration of 1 μg/mL or without LPS for 3 h in an FBS supplemented-culture medium.

### CAP treatment

A CAP apparatus (Pidi™, Sekisui Chemical, Osaka, Japan) was used with nitrogen as the feed gas. THP-1 cells stimulated with LPS were treated with CAP for 70 s at a flow rate of 1 L/min, 12 mm from the bottom of the well. After plasma irradiation, cells were incubated at 37°C in 5% CO_2_.

### RONS measurements

Hydrogen peroxide (H_2_O_2_) and nitrite (NO_2_^-^) concentrations were measured in the medium with or without cells following the CAP irradiation using the Amplite™ Colorimetric Hydrogen Peroxide Assay Kit (AAT Bioquest, Pleasanton, CA, USA) and the NO_2_/NO_3_ Assay Kit-FX (Fluorometric) 2,3-Diaminonaphthalene Kit (Dojindo, Tokyo, Japan), respectively. Absorbance and fluorescence were measured using the Varioskan LUX (Thermo Fisher Scientific).

### Cell viability assay

For CAP cytotoxicity evaluation, a 3-(4,5-dimethythizol-2-yl)-5-(3-carboxymethoxyphenyl)-2-(4-sulfophenyl)-2H-tetrazolium salt (MTS) assay and acridine orange (AO)/propidium iodide (PI) staining were conducted using the CellTiter 96^®^ AQueous One Solution Cell Proliferation Assay (Promega, Madison, WI, USA) and the Cyto3D Live-Dead Assay Kit (TheWell Bioscience, North Brunswick Township, NJ, USA), respectively. After CAP irradiation, MTS or AO/PI reagent was added to the wells, and absorbance or fluorescence was measured using the Varioskan LUX (Thermo Fisher Scientific).

### Reverse-transcription quantitative polymerase chain reaction (RT-qPCR)

RT-qPCR was performed to monitor Interleukin 6 (*IL6)* expression. After CAP irradiation, RNA was extracted from THP-1 cells and reverse-transcribed to synthesize cDNA using the CellAmp™ Direct RNA Prep Kit for RT-PCR (Takara Bio, Shiga, Japan). The PCR mixture was prepared using TB Green^®^ Fast qPCR Mix (Takara Bio), and RT-qPCR was performed using the QuantStudio^®^ 3 Real-Time PCR System (Thermo Fisher Scientific). Relative gene expression levels were calculated using the 2^−ΔΔCT^ method [15], and glyceraldehyde-3-phosphate dehydrogenase (GAPDH) was used as the internal control. The PCR primers used in this study are listed in Table 1.

**Table 1:** Primer sequences for RT-qPCR.

Target		sequence 5′→3′
*GAPDH*	forward	CATCTTCTTTTGCGTCGCC
	reverse	GTTAAAAGCAGCCCTGGTGAC
*IL6*	forward	GCTGATGGCCCTAAACAGA
	reverse	GGTGGTCGGAGATTCGTAG

### Enzyme-linked immunosorbent assay (ELISA)

Supernatants from LPS-stimulated THP-1 cell cultures were collected 12 and 24 h after CAP irradiation. IL6 in the medium was measured using an ELISA kit (R&D Systems, Minneapolis, MN, USA) according to the manufacturer’s instructions.

### RNA extraction and RNA-Seq

Total RNA was extracted from LPS-stimulated THP-1 cells using the RNeasy Plus Mini Kit (QIAGEN, Netherlands) 3, 6, 12, 18, and 24 h after 0 or 70 s of CAP irradiation. THP-1 cells irradiated and non-irradiated with CAP were named as CAP-treated and non-treated, respectively. RNA-Seq libraries were prepared using the poly(A) enrichment technique using the SMART Seq^®^ v4 Ultra^®^ Low Input RNA Kit for sequencing (Clontech, Shiga, Japan) from the total RNA. The libraries were sequenced with 2× 150-bp pair-end reads on a NovaSeq 6000 system (Illumina, San Diego, CA, USA). RNA-Seq data obtained in this study are available from the DNA Data Bank of Japan (DDBJ; http://www.ddbj.nig.ac.jp/) under the accession number DRA016810. DRAGEN Bio-IT Platform v3.6.3 (Illumina, USA) was used to map the obtained RNA-Seq data to the human reference genome (GRCh38.primary_assembly.genome.fa.gz) and calculate the gene expression levels. Genes were annotated using the GENCODE annotation file (gencode.v35.primary_assembly.annotation.gtf.gz).

### Western blotting

Nuclear proteins were extracted from THP-1 cells 3 h after CAP irradiation using the Nuclear Extraction Kit (Cayman Chemical, Ann Arbor, MI, USA). Protein samples were denatured with the 10× Bolt™ Sample Reducing Agent (Thermo Fisher Scientific) and the 4× Bolt™ LDS Sample Buffer (Thermo Fisher Scientific) separated by a Bolt™ 4 to 12% Bis-Tris, 1.0 mm, Mini Protein Gel (Thermo Fisher Scientific), and transferred to a polyvinylidene difluoride membrane using the iBlot 2 Dry Blotting System (Thermo Fisher Scientific). Specific protein bands were detected using anti-NRF2 (GTX103322, GeneTex, Irvine, CA, USA, 0.2 μg/mL) or anti-LAMIN B1 (GTX103292, GeneTex, 0.2 μg/mL) as primary antibodies, and HRP-conjugated anti-rabbit IgG (GTX213110-01, GeneTex, 0.02 μg/mL) as a secondary antibody using the iBind Automated Western System (Thermo Fisher Scientific). Protein bands were imaged using the FUSION-SOLO.7 S. EDGE V.070 (Vilber, France) and the SuperSignal™ West Femto Maximum Sensitivity Substrate (Thermo Fisher Scientific) as the chemiluminescent substrate and quantified using the ImageJ (ver. 1.53) [16].

### Statistical analysis

All quantitative data are shown as the mean and standard deviation from three independent experiments. Tukey’s or Dunnett’s tests were performed using the R software (ver. 4.2.0) to compare the multiple groups.

Differential expression analysis was performed using the R software (ver. 4.2.0) and the DEseq2 (ver. 1.36.0) to calculate normalized counts, fold changes (FC), and adjusted p-values (p-adj). Differentially expressed genes (DEGs) were defined when |log_2_(FC)| > 1 and p-adj < 0.05. Hierarchical clustering was performed with log_2_-transformed normalized counts using the Python (ver. 3.9.4) with the SciPy (ver. 1.6.3). Gene ontology (GO) enrichment and gene set enrichment analyses (GSEA) were conducted using the Python package gseapy (ver. 0.10.5), and terms or gene sets with a false discovery rate (FDR) < 0.05 were shown as results. Hallmark gene sets [17] were used for GSEA. The DoRothEA R package (ver. 1.8.0) [18] was employed to estimate TFs using the VIPER algorithm [19].

## Results

### Effects of CAP on cell viability, IL6 expression, and RONS accumulation

**Fig 1:**
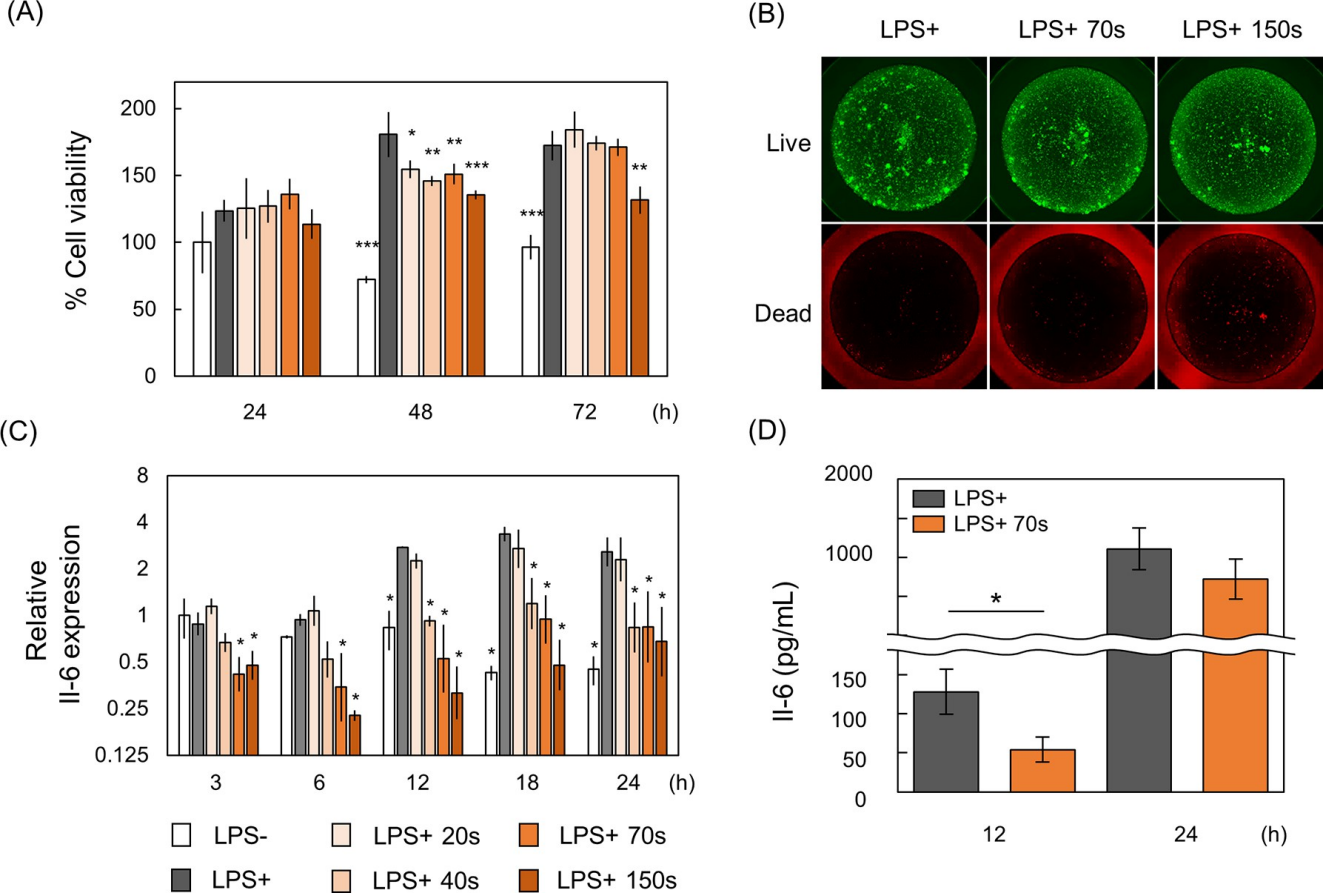
Cytotoxicity and IL6 expression following CAP irradiation. (A) Measurement of cell viability at 24–72 h post 20–150 s of CAP irradiation. Data represent the mean ± standard deviation of three independent experiments. *: p < 0.05, **: p < 0.01, ***: p < 0.001, compared to LPS+ using Dunnet’s multiple comparison test. (B) Live/dead cell staining performed 24 h after CAP irradiation. (C) *IL6* expression measured by qRT-PCR at 3–24 h post CAP irradiation, normalized to LPS+ as the control. Data represent the mean ± standard deviation of three independent experiments. *: p < 0.05, compared to LPS+ using Dunnet’s multiple comparison test. (D) Measurement of IL6 concentration in the culture medium using ELISA. *: p < 0.05, analyzed with Welch’s t-test.

The cell viability remained unaffected 24 h after CAP irradiation but declined after 48 h. Notably, cell viability decreased following 150 s of CAP irradiation at 72 h (Fig 1A). The number of dead cells showed no change after 70 s of CAP irradiation but slightly increased after 150 s (Fig 1B). *IL6* expression remained unaltered after 20 s of CAP irradiation; however, irradiation for 40 s or more significantly reduced expression levels at 12–24 h for 40 s of irradiation and at 3–24 h for 70 s and 150 s of irradiation (Fig 1C). CAP irradiation for 70 s led to a significant reduction in IL6 protein expression at 12 h (Fig 1D). These findings indicate that CAP irradiation exceeding 40 s exerts anti-inflammatory effects, while irradiation exceeding 150 s iscytotoxic. Thus, subsequent experiments utilized 70 s of CAP irradiation as it significantly suppressed IL6 expression without causing cytotoxic effects.

**Fig 2:**
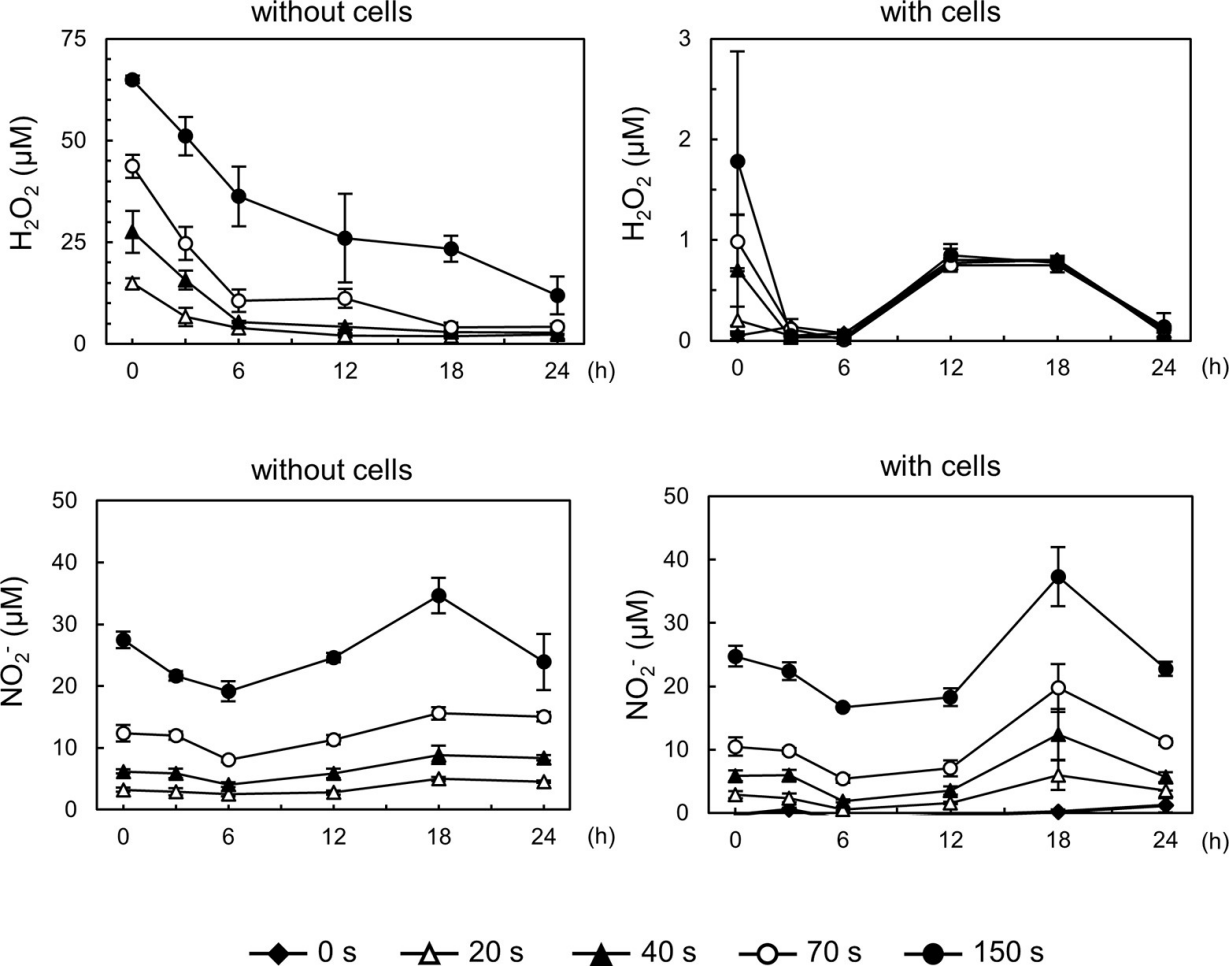
RONS accumulation in the CAP-irradiated medium. Concentrations of H_2_O_2_ and NO_2_^-^ in the medium with or without cells after 0–24 h following CAP exposure for each irradiation time. Data are presented as the mean ± standard deviation of three independent experiments.

The accumulation of RONS in the cell culture medium due to CAP treatment was quantified using a colorimetric assay. This assay precisely measured the concentrations of the long-lived reactive species H_2_O_2_ and NO_2_^-^. In the absence of cells, CAP irradiation for 20–150 s resulted in the production of 14.9–65.0 μM H_2_O_2_ and 3.2–27.5 μM NO_2_^-^, both of which gradually degraded over time. However, when cells were present, H_2_O_2_ rapidly decomposed, while NO_2_^-^ persisted even after 24 h (Fig 2).

## RNA-Seq data analyses

**Fig 3:**
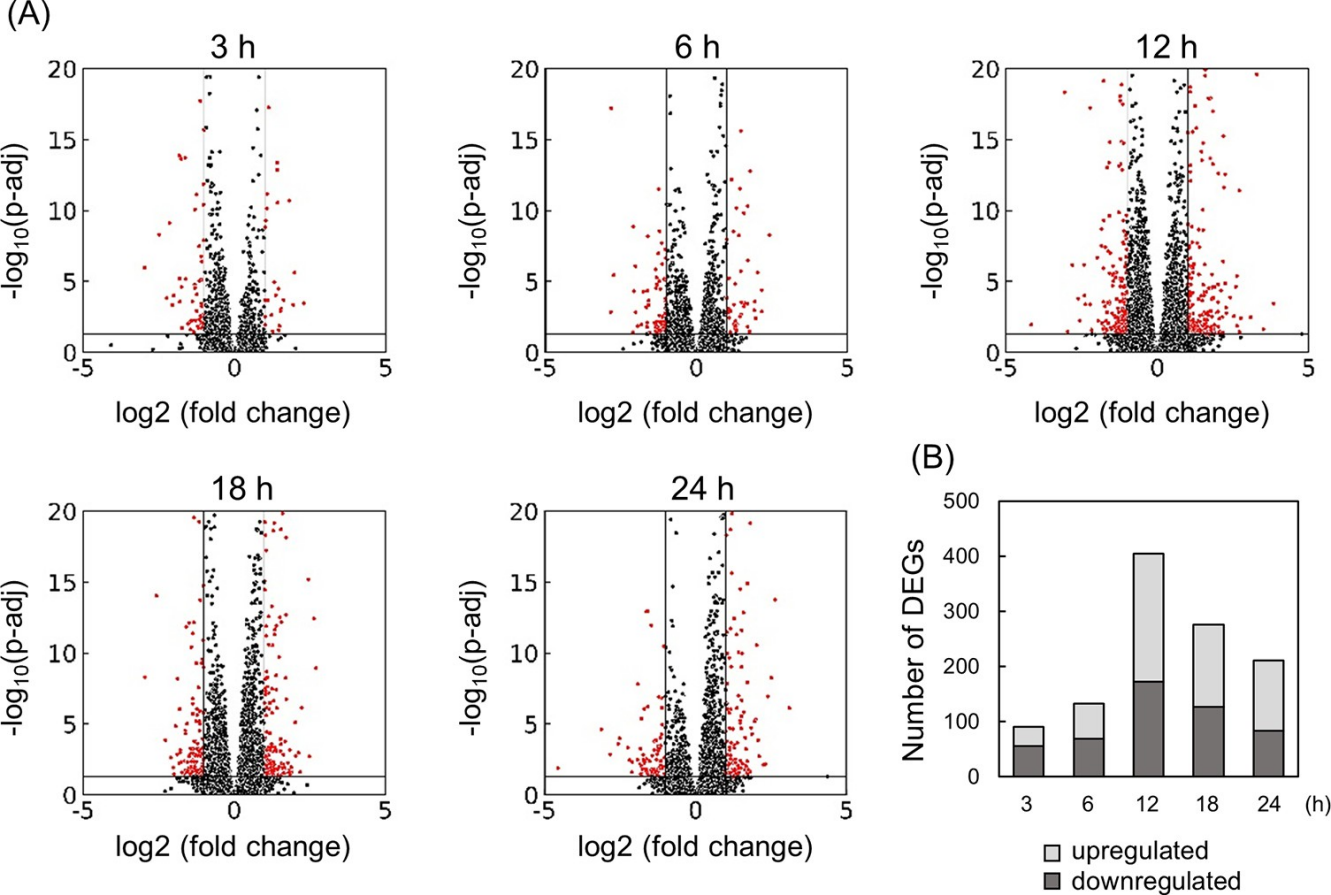
RNA-Seq analysis of THP-1 cells treated with CAP. (A) Volcano plot comparing the CAP-treated group with the non-treated group. Red points indicate DEGs, characterized by |log_2_(FC)| > 1 and p-adj < 0.05. (B) Stacked chart illustrating the numbers of upregulated (light grey) and downregulated (dark grey) DEGs.

To elucidate the anti-inflammatory mechanism of CAP, LPS-stimulated THP-1 cells were treated with and without CAP. RNA samples were extracted at 3, 6, 12, 18, and 24 h for RNA-Seq analysis. DEGs between the CAP-treated and the non-treated groups were analyzed. The count of DEGs reached a maximum of 405 after 12 h, indicating that CAP irradiation altered gene expression patterns (Fig 3A and 3B).

**Fig 4:**
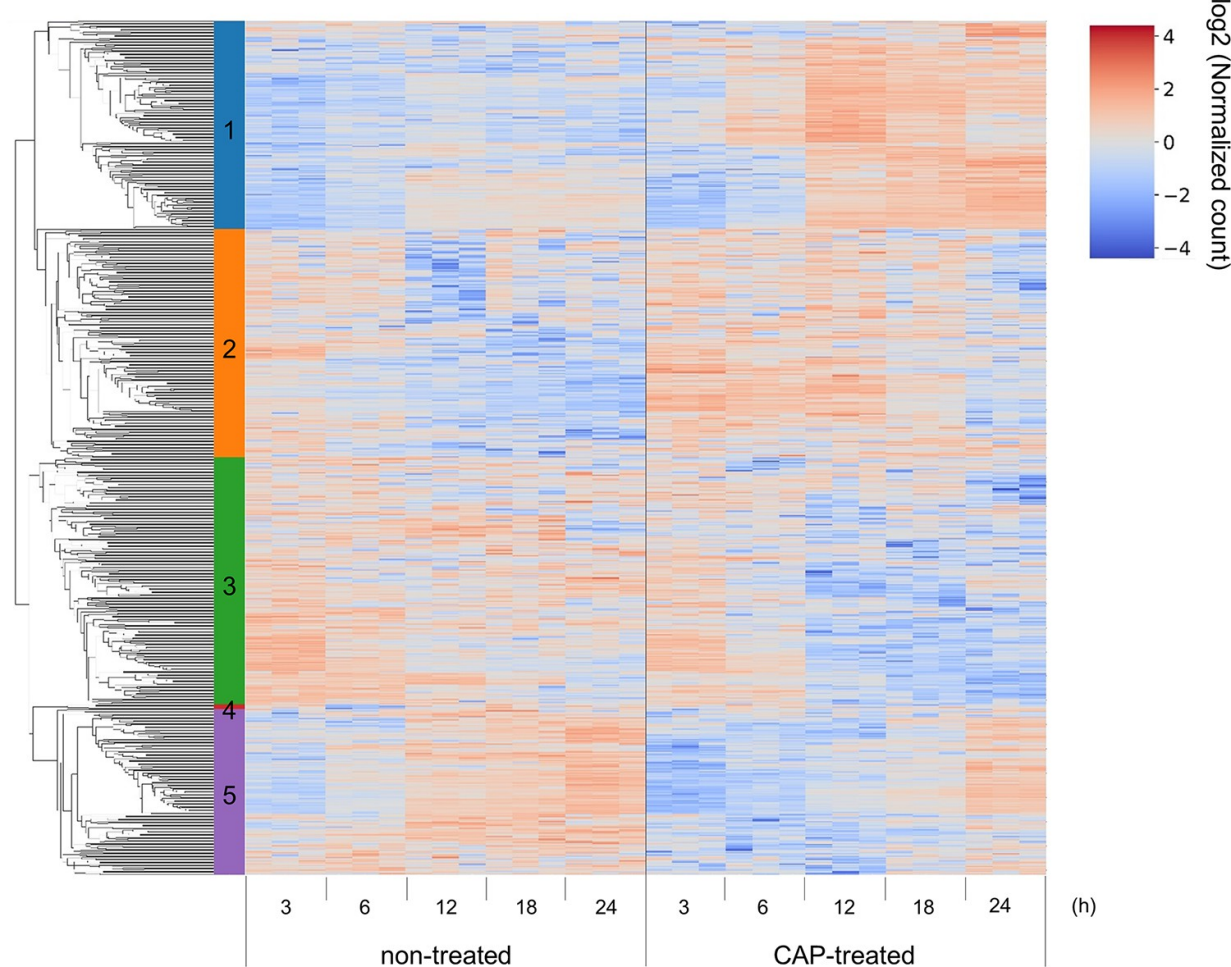
Hierarchical clustering of 740 DEGs at various time points. Input values consisted of log2-transformed normalized counts. The DEGs were classified into five clusters, comprising 180, 198, 214, 4, and 144 genes, respectively.

Hierarchical clustering analysis was conducted using 740 genes identified as DEGs at any given time to assess significant alterations in gene expression patterns (Fig 4). These genes were categorized into five clusters: cluster 1 included 180 genes that were activated 12 h after CAP irradiation (late activated), cluster 2 included 198 genes that were primarily activated 3–12 h after CAP (early activated), cluster 3 included 214 genes that were inhibited after 12 h (late inhibited), cluster 4 included only four genes, and cluster 5 included 144 genes that were inhibited before 18 h (early inhibited).

GO enrichment analyses of each cluster showed that “positive regulation of inflammatory response” and other inflammation-related terms were significantly enriched in cluster 1 (Table 2). No GO terms were enriched in cluster 2. Inflammation-related GO terms such as “positive regulation of interferon-gamma production” and “antigen processing and presentation of exogenous peptide antigen via MHC class II” were enriched in cluster 3. The major histocompatibility complex (MHC) class II-related genes were enriched in this cluster. Although cluster 1 showed a pro-inflammatory effect of CAP in the late phase, cluster 3 indicated that CAP simultaneously suppressed the inflammatory response. Cluster 5 also included inflammatory-related terms such as “positive regulation of cytokine production,” “inflammatory response,” “response to lipopolysaccharide,” “positive regulation of interleukin-6 production,” and “positive regulation of interleukin-1 beta production”. Hence, CAP suppressed inflammation during the early phase.

**Table 2:** Notable GO terms of biological processes in each cluster.

Cluster	Mode	GO Term (Biological Process)	Overlap	FDR	Genes
1	late activated	inflammatory response	12/230	0.0019	*TMIGD3*, *MMP25*, *CXCL8*, *C5AR1*, *CHI3L1*, *ADM*, *CCL1*, *CDL4*, *FCGR2B*, *THBS1*, *S100A8*, *CMKLR1*
		positive regulation of inflammatory response	6/89	0.0323	*OSM*, *S100A12*, *CCL1*, *ETS1*, *PLA2G7*, *S100A8*
3	late inhibited	positive regulation of interferon-gamma production	7/57	0.0031	*IL23A*, *SLC11A1*, *HLA-DPB1*, *IL12B*, *PTPN22*, *IL18R1*, *HLA-DPA1*
		antigen processing and presentation of exogenous peptide antigen via MHC class II	7/98	0.0126	*HLA-DMA*, *HLA-DMB*, *HLA-DPB1*, *HLA-DRA*, *HLA-DQA1*, *HLA-DPA1*, *HLA-DQB1*
		interleukin-23-mediated signaling pathway	3/9	0.0126	*IL23A*, *STAT4*, *IL12B*
5	early inhibited	positive regulation of cytokine production	13/335	0.0388	*CD86*, *HHLA2*, *CD80*, *LILRB1*, *LILRB2*, *LILRA2*, *SCIMP*, *LILRA5*, *TLR1*, *FGFR*, *IL6*, *POLR3G*, *CCR7*
		inflammatory response	11/230	0.0002	*TLR1*, *IL6*, *PTGIR*, *CCL8*, *FPR1*, *CCL2*, *CCR7*, *SIGLEC1*, *FPR2*, *NLRC4*, *CXCL13*
		response to lipopolysaccharide	9/159	0.0003	*CD86*, *CX3CR1*, *IL6*, *CD80*, *CD180*, *CCL2*, *LILRB1*, *LILRB2*, *CXCL13*
		positive regulation of interleukin-6 production	6/76	0.0010	*TLR1*, *IL6*, *LILRB2*, *LILRA2*, *SCIMP*, *LILRA5*
		positive regulation of interleukin-1 beta production	5/56	0.0024	*IL6*, *MNDA*, *NLRC4*, *LILRA2*, *LILRA5*

GSEA was conducted to examine the overall patterns of gene expression. The analysis revealed that the Normalized Enrichment Score (NES) for the "REACTIVE OXYGEN SPECIES PATHWAY" and "MTORC1 SIGNALING" gene sets were consistently elevated across all time points (Table 3). “TGF BETA SIGNALING” also showed significantly high NES values after 6 h. The NES values of “INFLAMMATORY RESPONSE” and “TNFA SIGNALING VIA NFKB” were significantly low at 3 h; however, they continued to increase at 6 h and beyond. These results not only confirm the anti-inflammatory effect of CAP but also suggest its pro-inflammatory effects after 12 h.

**Table 3:** Gene set enrichment analysis for each time point.

Gene Set		3 h	6 h	12 h	18 h	24 h	size
INFLAMMATORY RESPONSE	NES	-2.69	1.44	1.79	1.93	2.30	200
	FDR	0.000	0.056	0.009	0.001	0.000	
MTORC1 SIGNALING	NES	1.86	2.15	1.45	1.58	1.59	200
	FDR	0.004	0.003	0.036	0.015	0.009	
REACTIVE OXYGEN SPECIES PATHWAY	NES	1.95	2.81	2.39	2.28	2.28	49
	FDR	0.001	0.000	0.000	0.000	0.000	
TNFA SIGNALING VIA NFKB	NES	-2.57	2.11	2.23	2.27	2.23	200
	FDR	0.000	0.002	0.001	0.000	0.000	
TGF BETA SIGNALING	NES	1.27	1.87	2.02	2.26	2.12	54
	FDR	0.154	0.007	0.005	0.000	0.000	

To identify the key TFs that regulate anti-inflammatory responses, TFs activated and inhibited by CAP were estimated using DoRothEA [18], and the average FDR at all time points was calculated for all TFs (Table 4). According to the results of the average FDR at 3–24 h, regulatory factor X5 (RFX5), which regulates MHC class II-related gene expression [20], was predicted to be the most repressed TF, whereas NRF2, which regulates defense mechanisms against oxidative stress via ARE [21], was predicted to be the most promoted TF. BTB and CNC homolog 1 (BACH1), which acts antagonistically with NRF2 [22], and NRF1, which acts like NRF2 via ARE [23], were also activated.

**Table 4:** Top five estimated TFs activated or inhibited by CAP.

TF	3 h		6 h		12 h		18 h		24 h		FDR mean
	t value	FDR	t value	FDR	t value	FDR	t value	FDR	t value	FDR	
RFX5	-3.982	0.211	-9.903	0.040	-81.048	0.000	-8.116	0.145	-4.417	0.203	0.120
NRF2	6.752	0.118	12.425	0.033	10.256	0.069	5.125	0.194	4.640	0.203	0.123
BACH1	4.029	0.211	15.641	0.026	3.856	0.260	3.487	0.199	2.984	0.267	0.193
NRF1	3.131	0.272	3.558	0.216	4.278	0.233	6.321	0.145	2.949	0.267	0.227
JUNB	3.868	0.222	1.900	0.364	3.225	0.290	4.402	0.194	2.794	0.267	0.267

### Protein level alteration of the CAP-induced TFs

**Fig 5:**
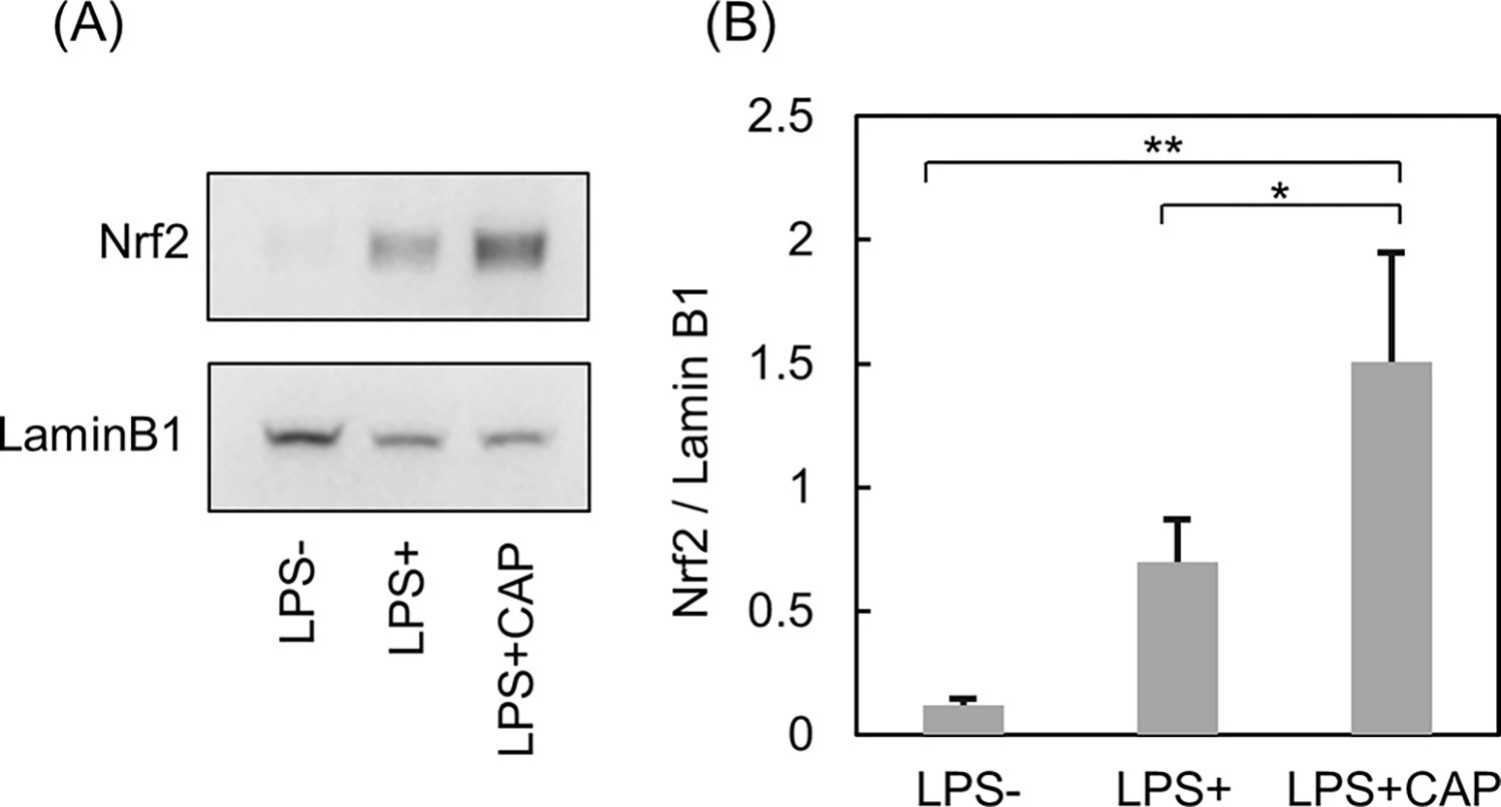
NRF2 localization in the nucleus of THP-1 cells. Western blotting images (A) and normalized quantification (B).LAMIN B1 was used as loading control. Data are presented as the mean ± standard deviation of three independent experiments. *p <0.05, **p <0.01; Tukey’s multiple comparison test was performed.

Since NRF2 was estimated to be the most activated TF following CAP irradiation, the nuclear transition of NRF2 was measured using western blotting. A significant increase of NRF2 accumulation in the nucleus of CAP-irradiated THP-1 cells was confirmed, suggesting that the nuclear transition of NRF2 was promoted by CAP irradiation (Fig 5).

## Discussion

Plasma medicine has emerged as a promising field of research for the treatment of various medical conditions, with a particular focus on wound healing and sterilization. Some studies have suggested the anti-inflammatory effects of CAP [5,9,10] and its application in the dental field, such as in periodontal disease [6]; however, how CAP inhibits inflammatory responses remains unclear. In the present study, we identified and confirmed the key TFs using transcriptome analysis to elucidate the anti-inflammatory mechanisms of CAP. THP-1, a human acute monocytic leukemia cell line, was used in the present study because monocytes play a central role in periodontal diseases, such as initiating inflammation upon contact with pathogens, inflammatory cytokine production, and T cell activation [24]. To induce inflammatory condition, Pg-derived LPS was employed since Pg is one of the most important pathogens in periodontitis [25,26], and Pg-LPS has been reported to induce inflammation in THP-1 [27,28]. Pg-derived LPS is known to induce inflammation mainly via TLR2 [29], and THP-1 expresses TLR2 more than TLR4 [30]. Proteins present in serum, such as LPS-binding protein, have a critical role in inducing interferon (IFN)-related genes [31], and IFN signaling is important in inflammasome activation against infection [32]. Thus, THP-1 was stimulated with Pg-LPS in an FBS-supplemented medium to mimic the *in vivo* environment.

CAP irradiation exhibited a dose-dependent suppression of *IL6* expression and increased cytotoxicity. The quantity of RONS generated at each dose was quantified. Non-irradiated cells did not produce H_2_O_2_ or NO_2_^-^ (Fig 2), indicating that the RONS were produced by the reaction between CAP and atmosphere and medium. H_2_O_2_ in the medium without cells was gradually degraded after 24 h. In contrast, H_2_O_2_ rapidly degraded in the medium with cells, indicating that THP-1 cells have a high capacity to decompose it. However, the NO_2_^-^ concentration was maintained for 24 h after irradiation, regardless of the presence or absence of cells. The anti-inflammatory effects of CAP are presumably attributed to NO_2_^-^ and its reduced form, nitric oxide, which regulates inflammation [33–35].

GO enrichment analysis revealed that CAP inhibited MHC class II-related gene and *IL23* expression. MHC class II, expressed on the membrane of antigen-presenting cells (APCs), such as monocytes, dendritic cells, and macrophages, mediates the presentation of exogenous antigens to CD4+ naïve T cells and promotes helper T cell differentiation [36,37]. CD4+ naïve T cells stimulated by APCs differentiate into subsets of helper T cells: Th1, which eliminates intracellular pathogens; Th2, which controls the immune response against extracellular parasites; Th17, which is responsible for mounting the immune response against extracellular bacteria and fungi; and regulatory T cells (Treg), which negatively regulates inflammation [37]. The involvement of Th1, Th2, and Th17 cells at periodontal disease sites has also been observed [38,39]. CD4+ naïve T cells differentiate into Th17 cells upon stimulation with IL6 and IL23 [40]. Th17 secretes IL17, which induces inflammation in other cells, such as fibroblasts and macrophages, and proliferates in the presence of IL23 [41,42]. IL17 is overexpressed in tissues with chronic inflammation, including in periodontitis, and the IL23 antagonist inhibits the *IL17* expression and inflammation [42,43]. Following CAP irradiation, our results revealed the inhibition of MHC class II-related genes, IL6 and *IL23*, suggesting that CAP attenuates Th17 cell differentiation and proliferation, leading to anti-inflammatory effects.

GSEA revealed that CAP activated transforming growth factor β (TGF-β) and mTORC1 signaling. TGF-β acts on CD4+ naïve T cells to inhibit the differentiation into Th1 and Th2 and promote differentiation into Tregs [44]. TGF-β also promotes CD4+ naïve T cell differentiation into Th17 with IL6 [37]; however, IL6 was downregulated by CAP, suggesting that CAP irradiation biases helper T cell population toward the Treg subset. Since pro-inflammatory effects have been observed by inhibiting mTORC1 [45,46], activating mTORC1 signaling using CAP may reduce inflammatory responses.

In the present study, NRF2 was the most upregulated TF, and its nuclear translocation was confirmed by western blotting. NRF2 activation via CAP was consistent with previous studies [13,14,47]. Under normal, non-stressful conditions, NRF2 undergoes ubiquitination by Kelch-like ECH-associated protein 1 (KEAP1) and subsequent proteasomal degradation. However, during oxidative stress, KEAP1 undergoes oxidation, leading to the release of NRF2. This release further enhances the expression of antioxidant genes regulated by AREs [21]. One such antioxidant gene is heme oxygenase 1 (*HO-1*), which generates the anti-inflammatory molecules carbon monoxide and biliverdin as byproducts of heme decomposition [48]. Additionally, NRF2 binds directly to regions proximal to the *IL6* and *IL1β* genes, inhibiting their expression [49]. Furthermore, activation of NRF2 inhibits the nuclear factor-kappa B pathway and ameliorates periodontitis in a rat model [50]. Furthermore, inhibition of IL6 and NRF2 nuclear translocation was confirmed in this study. Thus, NRF2 plays a pivotal role in suppressing inflammatory responses by activating antioxidant genes and inhibiting inflammatory cytokines.

In contrast, RFX5 was estimated to be the most downregulated TF. RFX5 binds to class II transactivator (CIITA) and other proteins and positively regulates MHC class II genes [51]. KEAP1 oxidation downregulates CIITA activity [52], suggesting that CAP suppresses MHC class II gene expression by affecting KEAP1 and CIITA. CAP treatment appeared to activate BACH1 and NRF1. BACH1 binds to AREs and competes with NRF2 for interaction [22]. BACH1 activation contributes to homeostasis-like effects by suppressing the NRF2 pathway. NRF1 overlaps with NRF2 [23], suggesting that it has anti-inflammatory and antioxidant effects.

However, the study had several limitations: the cell type was limited to monocytes, and no *in vivo* studies were conducted. Although NRF2 activation and nuclear transition promoted by CAP have been confirmed, KEAP1 oxidation induced by CAP and the KEAP1-CIITA pathway needs to be elucidated. Therefore, further research is necessary to reveal the inhibition of CD4+ naïve T cell differentiation into Th17 cell by CAP.

**Fig 6:**
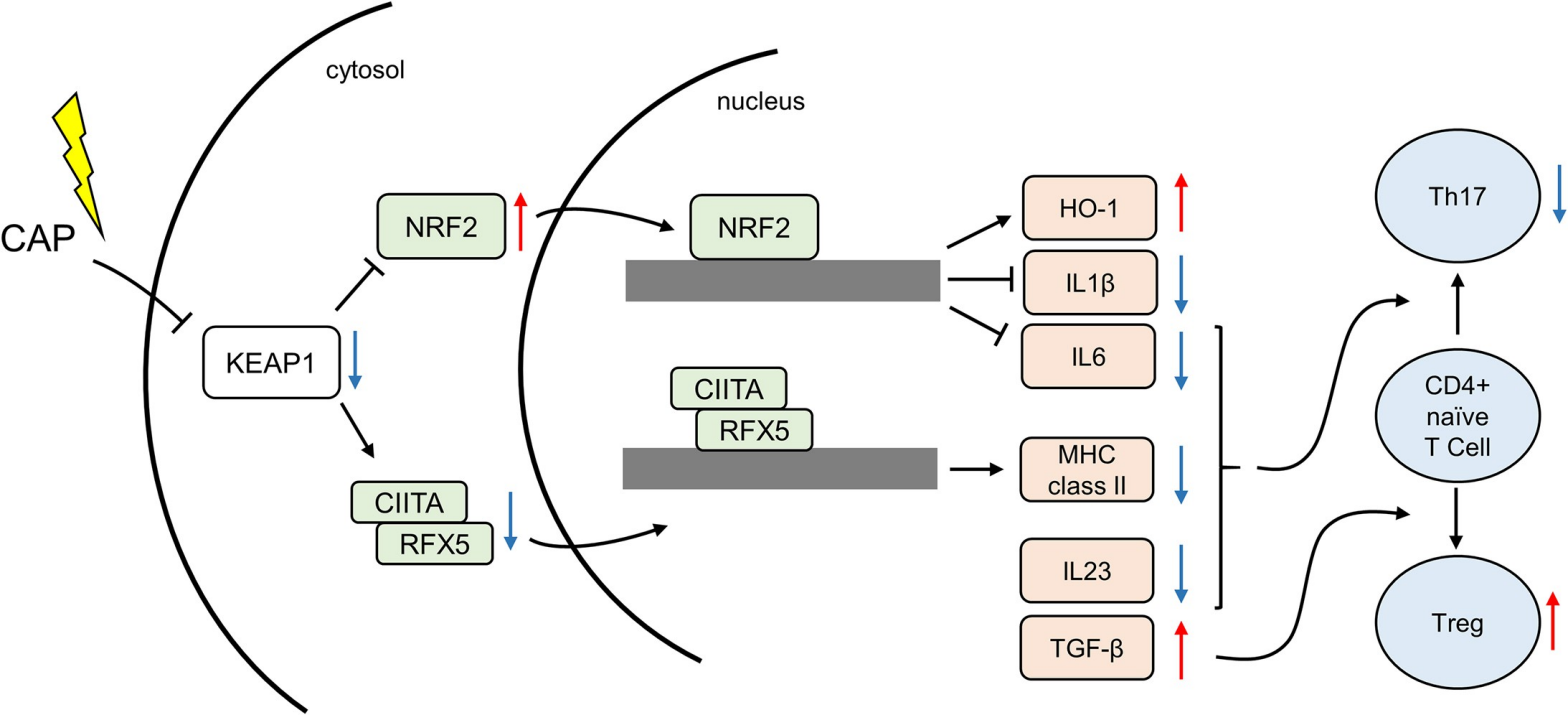
Schematic illustration depicting the proposed anti-inflammatory mechanism of CAP. Red and blue arrows represent the CAP-induced activation and suppression, respectively, in each pathway. CAP: Cold atmospheric plasma; KEAP1: Kelch-like ECH-associated protein 1; NRF2: Nuclear factor erythroid 2-related factor 2; CIITA: Class II transactivator; RFX5: Regulatory factor X5; HO-1: Heme oxygenase 1; IL1β: Interleukin 1β; IL6: Interleukin 6; MHC class II: Major histocompatibility complex class II; IL23: Interleukin 23; TGF-β: Transforming growth factor β; Treg: Regulatory T cell.

Collectively, our findings demonstrated that the NRF2 pathway is involved in mediating the anti-inflammatory effects of CAP, potentially by suppressing pro-inflammatory genes, such as *IL6*, and upregulating anti-inflammatory genes, such as *HO-1*. Additionally, CAP downregulated RFX5, a regulator of MHC class II expression, and *IL23* while promoting TGF-β signaling. These findings suggest that CAP attenuated Th17 cell activity and promoted Treg cell differentiation, thus contributing to its anti-inflammatory effects (Fig 6).

## Supporting information

S1 Raw images (PDF)
