# Correction: Anti-inflammatory effects of cold atmospheric plasma irradiation on the THP-1 human acute monocytic leukemia cell line

**DOI:** 10.1371/journal.pone.0299329

**Published:** 2024-02-16

**Authors:** Ito Hirasawa, Haruka Odagiri, Giri Park, Rutvi Sanghavi, Takaya Oshita, Akiko Togi, Katsunori Yoshikawa, Koji Mizutani, Yasuo Takeuchi, Hiroaki Kobayashi, Sayaka Katagiri, Takanori Iwata, Akira Aoki

In the Table 1, the primer sequences of the forward (GCTGATGGCCCTAAACAGA) and reverse (GGTGGTCGGAGATTCGTAG) for *IL6* are incorrect. The correct sequences for *IL6* are as follows: forward (CCACTCACCTCTTCAGAACG) and reverse (CATCTTTGGAAGGTTCAGGTTG). Please see the correct Table 1 here.

**Table 1:** Primer sequences for RT-qPCR.

Target		sequence 5′→3′
*GAPDH*	forward	CATCTTCTTTTGCGTCGCC
	reverse	GTTAAAAGCAGCCCTGGTGAC
*IL6*	forward	CCACTCACCTCTTCAGAACG
	reverse	CATCTTTGGAAGGTTCAGGTTG
