# Supplementary material for: Anti-inflammatory effects of cold atmospheric plasma irradiation on the THP-1 human acute monocytic leukemia cell line
Source: PLoS One. 2023 Oct 18;18(10):e0292267. doi: 10.1371/journal.pone.0292267 (PMC10584116; doi:10.1371/journal.pone.0292267)
Supplement: S1 Raw images — (PDF) [file pone.0292267.s001.pdf]

Anti-inflammatory effects of cold atmospheric plasma irradiation on the THP-1 human acute monocytic leukemia cell line

S1 raw images

Western blotting images were taken with FUSION-SOLO.7 S. EDGE V.070 (Vilber, France), using SuperSignal™ West Femto Maximum Sensitivity Substrate (Thermo Fisher Scientific) as the chemiluminescent substrate.

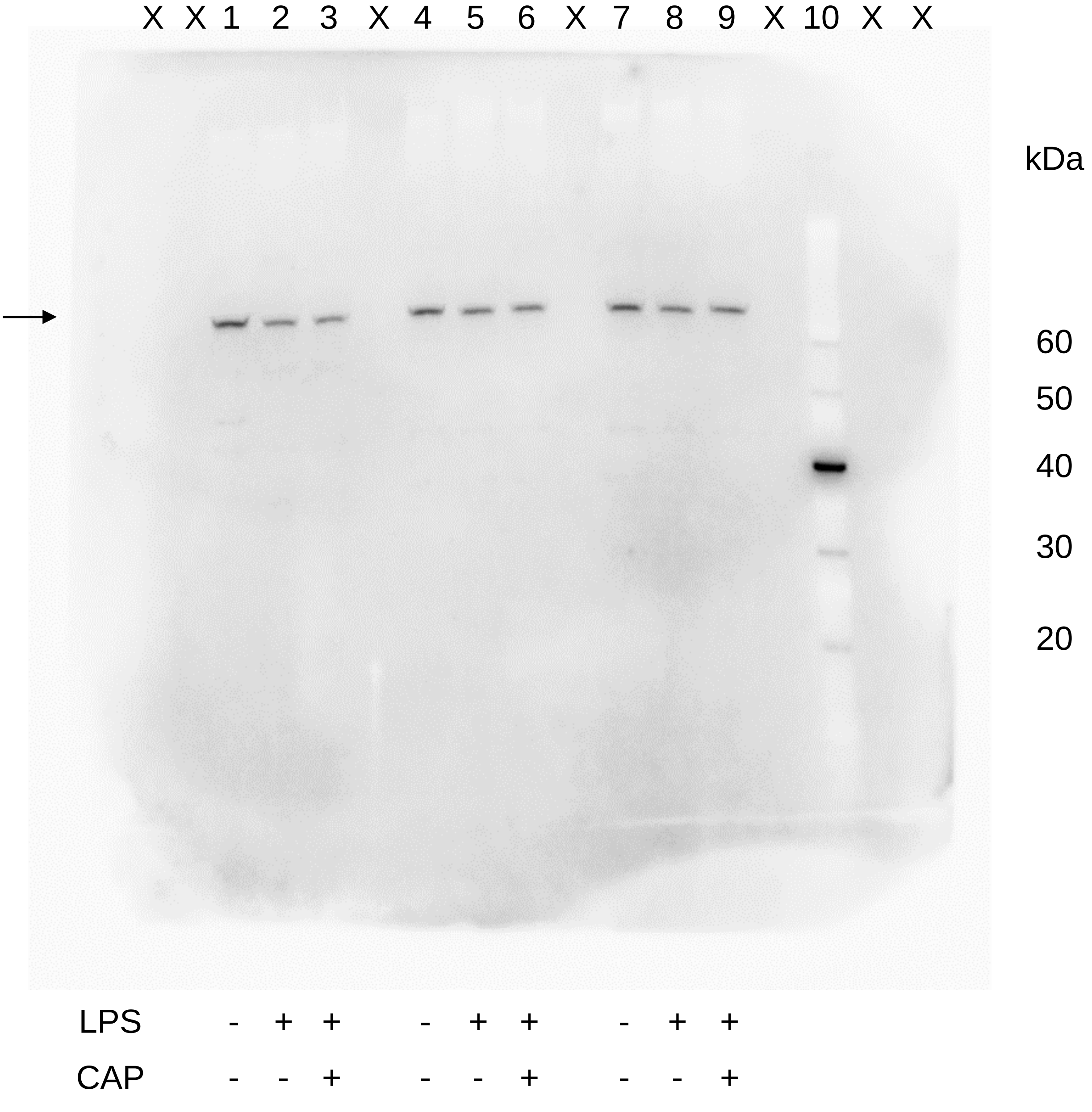

Fig S1A. Western blotting of LAMIN B1.
Lanes 1,4, and 7: negative control. Lanes 2,5, and 8: LPS-treated control. Lanes 3,6, and 9: CAP-treated samples. Lane 10: Molecular weight marker. Black arrow indicates the bands of LAMIN B1. The intensity of the bands indicated by black arrow in lane 1–9 was quantified using ImageJ. Lanes 1–3 were cropped and used to generate Fig 5.

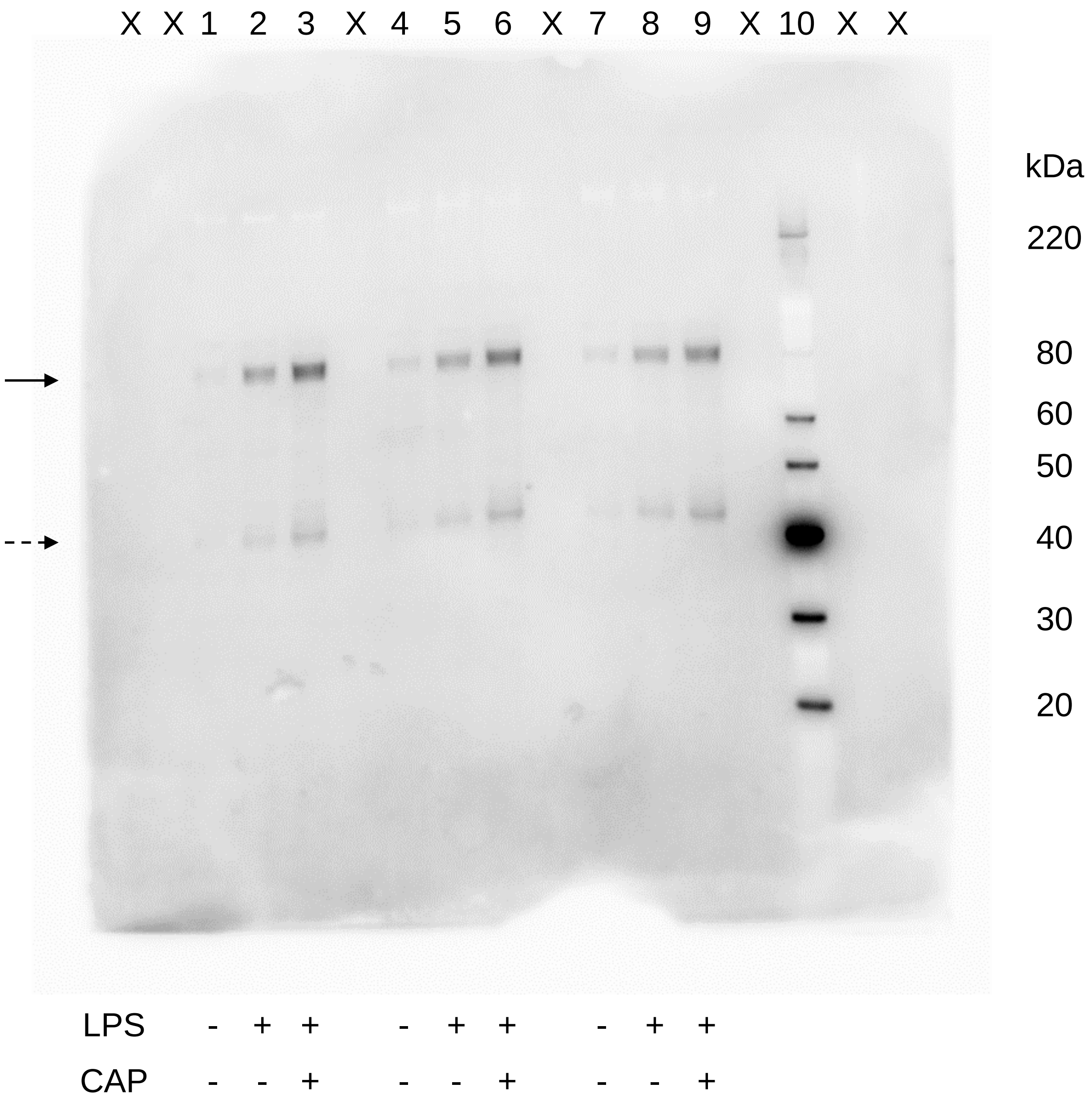

Fig S1B. Western blotting of NRF2.

Lanes 1,4, and 7: negative control. Lanes 2,5, and 8: LPS-treated control. Lanes 3,6, and 9: CAP-treated samples. Lane 10: Molecular weight marker. Black arrow indicates the bands of NRF2. Dotted-arrow might indicate the bands of degraded-NRF2. The intensity of the bands indicated by black arrow in lane 1–9 was quantified using ImageJ. Lanes 1–3 were cropped and used to generate Fig 5.
